# Cardiovascular Benefits of Resistance Training in Patients With Heart Failure With Reduced Ejection Fraction: A Systematic Review

**DOI:** 10.7759/cureus.47813

**Published:** 2023-10-27

**Authors:** Anirudh Danduboyina, Binay K Panjiyar, Saatvika R Borra, Sourav S Panicker

**Affiliations:** 1 Department of Internal Medicine, Gandhi Medical College, Hyderabad, IND; 2 Department of Internal Medicine, California Institute of Behavioral Neurosciences & Psychology, Fairfield, USA; 3 Department of Internal Medicine, Jawaharlal Nehru Medical College, Belagavi, IND; 4 Department of Internal Medicine, Dr. D. Y. Patil Medical College, Hospital and Research Centre, Pune, IND

**Keywords:** combined exercise therapy, aerobic training, heart failure with reduced ejection fraction, exercise training, resistance training

## Abstract

Cardiovascular diseases pose a serious problem for health globally. Among these, congestive heart failure is the leading cause of mortality and morbidity worldwide. According to the recent census, heart failure contributes to a huge financial burden annually. Exercise therapy is an integral part of the non-pharmacological management of heart failure. Due to the availability of various types of exercise therapies and rapid advancements in the existing evidence, it is often challenging to prescribe an appropriate exercise program. Although there is unequivocal evidence supporting the cardiovascular benefits of aerobic therapy, the incorporation of resistance training into exercise regimens should also be encouraged due to its effects on muscular endurance and ameliorating skeletal myopathy in heart failure.

In this study, we used a systematic literature review (SLR) approach to give an overview of the current literature and highlight the cardiovascular benefits of resistance training, alone or in combination with aerobic training. We reviewed articles from well-recognized journals published between 2013 and 2023, finally narrowing down to nine selected papers for a thorough analysis. The inclusion criteria comprise studies dealing with heart failure with reduced ejection fraction (HFrEF), resistance training alone or in combination with aerobic therapy, and studies available for free in either the PubMed or Google Scholar databases. The systematic review revealed that resistance training in combination with aerobic therapy has greater cardiovascular benefits than either resistance or aerobic therapy alone. A few unique approaches, like periodic intermittent muscular exercise (PRIME) and super circuit training (SCT), have demonstrated an improvement in cardiac and non-cardiac clinical outcomes compared to conventional exercise therapies. Moreover, various factors, like lack of motivation and lack of time, contribute to poor adherence to exercise therapy. Approaches like telerehabilitation and designing exercise regimens with activities that patients enjoy have led to improvements in long-term adherence rates. Nevertheless, further exploration and research by conducting randomized controlled trials on a larger scale is essential to explore the potential of resistance training in the rehabilitation of patients with heart failure with reduced ejection fraction and to develop the most effective exercise therapy.

## Introduction and background

Chronic heart failure (CHF) is a clinical syndrome that remains the leading cause of mortality and morbidity worldwide [1]. Its prevalence, according to the 2023 American Heart Association Statistical Update, is estimated to be approximately 2.3% of the total US population [2]. The total cost of HF was about 31 billion dollars in the USA in 2012, which is set to reach up to 70 billion dollars by 2030 [3]. Heart failure is not a single pathological entity but a clinical syndrome comprising cardinal symptoms (e.g., shortness of breath, ankle swelling, and fatigue) that may be associated with signs (e.g., elevated jugular venous pressure, pulmonary crackles, and peripheral edema). It is due to a structural and/or functional abnormality of the heart that leads to elevated intracardiac pressures and/or inadequate cardiac output at rest and/or during exercise [4]. Traditionally, HF has been divided into distinct phenotypes based on the measurement of left ventricular ejection fraction (LVEF). Patients with LVEF of <40% have a significant reduction in LV systolic function and are categorized under heart failure with reduced ejection fraction (HFrEF). Those with LVEF between 41% and 49% who have mildly reduced LV systolic function are labeled as having heart failure with mid-range ejection fraction (HFmrEF), and those with symptoms and signs of HF, with evidence of structural and/or functional cardiac abnormalities and/or raised natriuretic peptides (NPs), and with an LVEF >50%, are included under heart failure with preserved ejection fraction (HFpEF).

Heart failure has a wide range of etiologies, like coronary heart disease, hypertension, valve disease, arrhythmias, cardiomyopathies, congenital heart disease, infections, drug-induced, infiltrative, storage disorders, endomyocardial diseases, pericardial disease, metabolic diseases, and neuromuscular disease [2]. In Western and developed countries, coronary artery disease (CAD) and hypertension are predominant causative factors [1]. Heart failure is managed with both pharmacological and non-pharmacological aspects. One of the most important aspects of non-pharmacological modalities of treatment is exercise therapy. The majority of the evidence suggests that the addition of exercise therapy to the treatment regimen leads to remarkable improvement in the health of patients with stable HF in New York Heart Association (NYHA) functional classes II or III, and no significant benefit has been observed in NYHA functional class IV [5]. The chief components of exercise therapy in cardiac rehabilitation are aerobic therapy (either high-intensity interval training (HIIT) or moderate continuous aerobic therapy (MCAT)), resistance training (RT), and inspiratory muscle training (IMT). Exercise training reestablishes equilibrium through its neurohumoral and anti-inflammatory effects and improves endothelial and skeletal muscle function [6]. The beneficial neuro-humoral effects of exercise training include the reversal of autonomic dysfunction by shifting sympathetic to vagal activity and lowering the circulating levels of neurohormones like angiotensin II, aldosterone, vasopressin, and natriuretic peptides [7, 8]. The anti-inflammatory effects of regular exercise training have been demonstrated in experimental models where exercise training led to an increase in anti-inflammatory cytokines and a decrease in plasma levels of inflammatory cytokines like tumor necrosis factor (TNF) and interleukin-6 (IL-6) through modulating macrophage and lymphocyte function [6]. Furthermore, experimental studies suggest that exercise training increases oxidative enzyme activity and mitochondrial content, thereby improving oxygen utilization and reducing oxidative stress [9]. However, the addition of exercise therapy has not demonstrated a significant improvement in mortality or the rate of hospitalization [10]. Lines of evidence demonstrate that HIIT is more effective in reversing left ventricular remodeling and improving cardiac output, endothelial function, maximum oxygen consumption (VO_2_ max), and quality of life compared with MCAT [11,12]. Moreover, the addition of inspiratory muscle training to aerobic or resistance training exhibited improved inspiratory muscle strength, resting heart rate, heart rate reserve, and health-related quality of life, as evidenced by the increased total score in the Minnesota Living with Heart Failure Questionnaire [13].

Although there is astounding evidence supporting the cardiovascular benefits of aerobic therapy in patients with HFrEF, notwithstanding the obvious implications, the participation rate is lower than expected among Medicare beneficiaries [14]. On the other hand, the effects of resistance training on the cardiovascular health of patients with HFrEF have been explored to a relatively lesser extent. In this light, we would like to conduct a meta-analysis to further study the cardiovascular benefits of resistance training in patients with HFrEF and their clinical and statistical significance.

## Review

**Figure 1:**
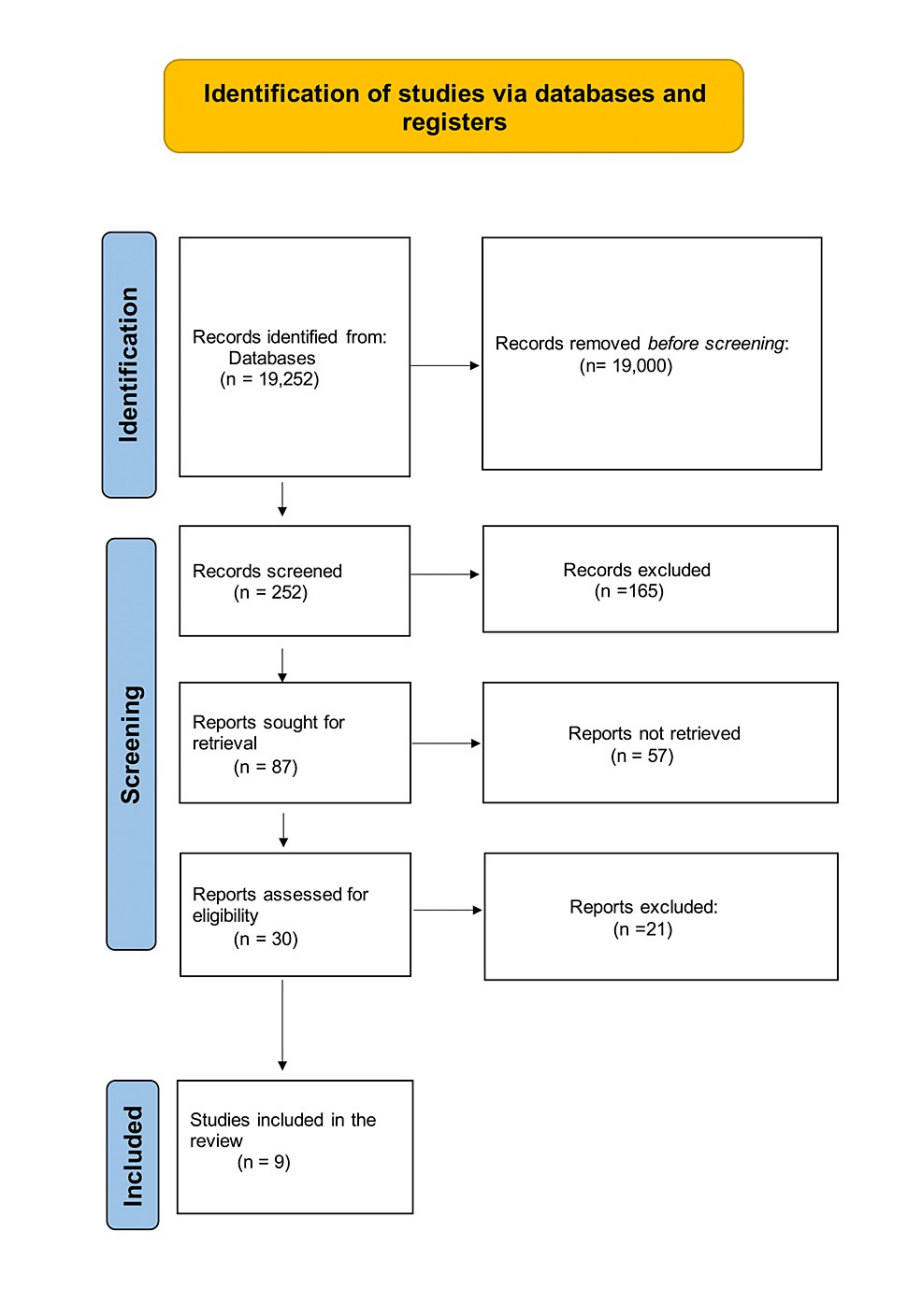
PRISMA flow diagram illustrating the search strategy and study selection process for the systematic review PRISMA: Preferred Reporting Items for Systematic Reviews and Meta-Analyses

The review deals with clinical studies concerning the role of resistance training in the cardiovascular health of patients with heart failure with reduced ejection fraction. We excluded animal studies and publications that only dealt with aerobic exercise therapy. The review follows the guidelines for Preferred Reporting Items for Systematic Reviews and Meta-Analyses (PRISMA) [15] as displayed in Figure 1 and exclusively uses data collected from published papers, eliminating the need for ethical approval.

Systematic literature search and study selection

We implemented a thorough search for relevant publications by using PubMed and Google Scholar. We specifically searched for studies mentioned in review papers, editorials, and commentaries on PubMed. However, we continued searching for additional studies fulfilling our inclusion criteria.

Inclusion and exclusion criteria

We applied certain criteria for including and excluding participants to satisfy our study goals. Our criteria are summarized in Table 1.

**Table 1:** Inclusion and exclusion criteria implemented during the literature search process NYHA: New York Heart Association

S.NO.	INCLUSION CRITERIA	EXCLUSION CRITERIA
a)	Human studies	Animal studies
b)	Age >13 years	Age<= 13 years
c)	Free papers	Papers that needed to be purchased
d)	Studies involving resistance or combined therapy	Studies involving only aerobic or inspiratory muscle training
e)	Patients with heart failure of NYHA grades I, II, or III	Patients with heart failure of NYHA grade IV
f)	English text	Non-English texts
g)	Gender: All	
h)	Heart failure with reduced ejection fraction i.e., <40% and heart failure with mildly reduced ejection fraction (EF) i.e., EF= 41-49%	
i)	Published from 2013 to 2023	

Search strategy

The population, intervention/condition, and outcome (PICO) criteria were applied to implement a thorough literature review. The search was conducted on databases such as PubMed and Google Scholar libraries with appropriate keywords like "heart failure", "reduced ejection fraction", and "resistance training". The medical subject heading (MeSH) approach for PubMed and Google Scholar, as exhibited in Table 2, was utilized to develop a comprehensive search strategy.

**Table 2:** Search strategy, search engines used, and the number of results

DATABASE	SEARCH STRATEGY	SEARCH RESULTS
PubMed	Cardiovascular benefits OR heart failure OR reduced ejection fraction OR resistance training, AND (2013/08/13 to 2023/08/23)	195,127
	Cardiovascular benefits AND resistance training, OR heart failure OR reduced ejection fraction AND (2013/08/13 to 2023/08/23)	161,433
	Heart failure AND resistance training AND reduced ejection fraction AND (2013/08/13 to 2023/08/23)	52
Google Scholar	Cardiovascular benefits of resistance training in patients of heart failure with reduced ejection fraction AND (2013 – 2023)	19,200

Quality appraisal

To ensure the reliability of the selected papers, we utilized various quality assessment tools. We applied the PRISMA checklist for systematic reviews, meta-analysis, and Cochrane bias tool assessment for randomized clinical trials. Non-randomized clinical trials were evaluated using the Newcastle-Ottawa tool scale. To avoid any uncertainty in classification, we utilized the scale for the assessment of narrative review articles (SANRA) to examine the article’s quality (Table 3).

**Table 3:** Quality-appraisal tools used PRISMA: Preferred Reporting Items for Systematic Reviews and Meta-Analyses; non-RCT: non-randomized control trial

QUALITY-APPRAISAL TOOLS IMPLEMENTED	QUALITY OF THE STUDY
Cochrane Bias Tool Assessment	Randomized control trials
Preferred reporting items for systemic reviews and meta-analyses (PRISMA) checklist	Systematic review
PRISMA checklist	Meta-analyses
Newcastle- Ottawa tools	Non-RCT and observational studies
Scale for the assessment of non-systematic review articles (SANRA) checklist	Any other without a clear method section

Results

After applying the above-mentioned search strategies to two databases, i.e., PubMed and Google Scholar, we extracted 19,252 articles. We then applied specific criteria and eventually eliminated 19,000 articles. From the remaining 252 papers, we chose not to utilize 165 of them due to duplicates and unsatisfactory titles and abstracts. Of the remaining 87 articles, 57 were not retrieved. We keenly went through the leftover 30 papers, and 21 of their contents did not fulfill our inclusion criteria. These nine articles are included in our final systematic review. Table 4 provides a detailed description of each article included in the study.

**Table 4:** Summary of the results of the selected papers CAT: continuous aerobic therapy; SCT: super circuit training; HFrEF: heart failure with reduced ejection fraction; PRIME: periodic intermittent muscle exercise; COMBO: combined moderate-intensity aerobic and resistance training; VO_2_ MAX: maximum oxygen uptake

S.NO	Author/Year	Country	Study design	Database used	conclusion
1.	Piepoli et al., 2013 [6].	Netherlands	Narrative review	PubMed	Aerobic therapy demonstrates a greater increase in VO_2_ max than resistance therapy.
2.	Springer et al., 2017 [16].	Germany	Narrative review	PubMed	The most effective strategy for managing sarcopenia in elderly patients with heart failure is resistance exercise.
3.	Giuliano et al., 2016 [17].	Australia	Randomized control Trial	Google Scholar	Resistance training as a single therapy can improve muscle strength, aerobic capacity, and quality of life in patients with heart failure with reduced ejection fraction.
4.	Esposito et al., 2018 [18].	USA	Randomized control Trial	PubMed	Following knee extensor training, the quadriceps muscles in patients with HFrEF demonstrated a significant increment in muscle fiber cross-sectional area, mitochondrial density, percentage area of type-1 muscle fibers, and a significant decrease in leg vascular resistance and noradrenaline spillover.
5.	Vuckovic et al., 2013 [19].	USA	Systematic review	PubMed	Resistance training in patients with HFrEF has improved endothelium-dependent vasodilation.
6.	Wang et al., 2022 [20].	China	Randomized controlled trial	PubMed	Resistance training when combined with simvastatin therapy demonstrated improved cardiac function, performance in a six-minute walk test, and a decrease in inflammatory markers, mitochondrial injury markers, and adverse cardiac events when compared with simvastatin therapy alone.
7.	Alshamari et al., 2023 [21].	Greece	Randomized controlled trial	Google Scholar	The combination therapy group demonstrated higher improvement in one repetition maximum test, muscular endurance, and peak work rate.
8.	Giuliano et al., 2020 [22].	Australia	Randomized control trial	PubMed	Implementing the PRIME training strategy prior to COMBO has shown greater improvement in aerobic capacity than COMBO in older patients with HFrEF.
9	Dor-Hiam et al., 2018 [23].	Israel	Randomized control trial	PubMed	When compared to the CAT group, the SCT group exhibited significantly greater improvement in the diastolic function of the heart, rate pressure product, metabolic equivalents, and the physical component of the general health-related quality of life questionnaire.

Discussion

Exercise plays a crucial role in the rehabilitation of patients with heart failure with reduced ejection fraction. In CHF, various anti-oxidative and catabolic processes, i.e., wasting of myofibrillar proteins of the diaphragm and quadriceps muscles, lead to exercise intolerance, ventilatory insufficiency, and chronotropic incompetence [16]. Furthermore, a surge in inflammatory cytokines in CHF leads to anorexia, and malnutrition and further contributes to sarcopenia and exercise intolerance [16]. The resistance training promotes endothelium-dependent vasodilation, thereby re-establishing the perfusion of the muscles (QO2) and promotes growth of muscle fiber cross-sectional area, mitochondrial density, percentage area of type-I muscle fibers, a significant decrease in leg vascular resistance and noradrenaline spillover, and improves muscular endurance and muscle strength, thereby increasing exercise tolerance [17-19]. One study in China demonstrated that resistance training in combination with Simvastatin therapy over a period of six months in patients with heart failure with reduced ejection fraction led to improved cardiac function, as evidenced by decreased interventricular septal thickness (IVST), left ventricular diastolic diameter (LVDD), and increased LVEF; improved mitochondrial function, i.e., increased Janus kinase/signal transducers and activators of transcription (JAK/STAT) 3 mRNA levels; decreased serum inflammatory markers such as C-reactive protein (CRP), galectin-3, and IL-6; improved performance in the six-minute walk test; and a relatively lower incidence of adverse cardiac events when compared to simvastatin therapy alone [20]. Resistance training, possibly combined with adequate protein intake, is the most effective intervention to prevent sarcopenia in older patients with HFrEF [16].

There are four main types of resistance training: isometric exercise, dynamic resistance exercise, combined isometric/dynamic exercise, and isokinetic exercise. Isometric exercise involves the contraction of a muscle without a change in muscle length, which significantly increases intramuscular pressure, leads to complete obstruction of blood flow to the contracting muscle, and contributes to a higher increase in mean arterial pressure for given oxygen consumption than equivalent dynamic exercise. On the other hand, dynamic resistance training, which is the most recommended mode of resistance training for patients with heart failure, allows a change in muscle length and thereby does not lead to an excessive increase in intramuscular pressure or complete obstruction of blood flow to the contracting muscle. Consequently, systolic blood pressure and pressure load on the heart do not increase as much as isometric exercise. The appropriate recommendation of resistance training for patients with heart failure has to be done according to the NYHA grade. For patients with NYHA I, four to five exercises, each with one or two sets of six to 10 repetitions per set with an intensity of 50%-60% of one repetition maximum (one RM), 15-20 min per day with a frequency of two days per week as a supplement to aerobic training, are recommended. For patients with NYHA grade II-III, three to four exercises, each with one to two sets of four to six repetitions per set with an intensity of 40%-50% of one rep maximum, 12-15 min per day with a frequency of one to two days per week as a supplement to aerobic training, are recommended. Whole-body training can be prescribed to patients belonging to NYHA grade I only after exclusive segmental training in the early months if tolerated well. Whereas in patients of NYHA grade II-III, whole-body training is rarely recommended. Patients with heart failure of NYHA grade IV should be deferred from implementing resistance training in their exercise regimen [24].

Nevertheless, when compared to resistance training, traditional aerobic therapy has shown better improvement in the VO_2_ max, which is the key prognostic factor in HF [6]. Hence, the approach of combining resistance therapy with aerobic therapy is widely being studied to test its superiority over traditional resistance or aerobic therapy alone. Resistance therapy can be integrated with aerobic therapy in several ways. In a randomized controlled trial, the addition of resistance training to HIIT has not only demonstrated similar improvement in resting oxygen consumption, peak oxygen consumption, predicted peak oxygen consumption, peak work rate (WR peak), heart rate recovery (HRR), and work rate at anaerobic threshold (WR at AT) to that of HIIT alone, but the combination therapy exhibited greater improvement in one RM test and muscular endurance of the chest muscles when compared to HIIT therapy alone [21]. Periodic intermittent muscle exercise is a variant of resistance training that essentially involves eight exercises, namely: chest press, leg press, seated row, triceps pushdown, latissimus dorsi pulldown, upright row, hack squat, and calf raises. Despite the absence of traditional aerobic exercise in the training protocol, the implementation of PRIME for four weeks followed by four weeks of combination therapy in older adults led to a greater increase in VO_2_ max than eight weeks of combination therapy alone [22]. Dor-Haim, H., compared the benefits of the continuous aerobic training (CAT) group with the SCT group. Each SCT set included one resistance training set followed by three minutes of aerobic exercise ranging from moderate to high intensity and a resting period. This sequence was repeated eight times. The resistance training consisted of eight different exercises, such as horizontal rowing, chest press, leg press, shoulder press, leg extension, lateral pulldown, leg flexion, and assisted squat. Unlike the continuous aerobic therapy group, which did not exhibit a significant change in cardiac function, the super circuit training group demonstrated a significant improvement in E/e', i.e., the ratio of the peak early mitral inflow velocity (E) over the early diastolic mitral annular velocity (e'), which is a measure of the diastolic function of the heart and the ejection fraction. Among the non-cardiac clinical outcomes, the super circuit training group demonstrated greater improvement than continuous aerobic therapy in metabolic equivalents (MET), rate pressure product (RPP), and the physical component of the quality-of-life scale (PCS) [23].

Despite the existence of volumes of evidence supporting the cardiovascular benefits of exercise therapy in patients with heart failure, 40%-91% of patients do not participate in regular exercise, with the percentage dropout ranging from 33%-56% during the actual program [25,26]. Various factors that contribute to poor adherence include age, low level of education, being socio-economically disadvantaged, lack of motivation for exercise, lack of time, laziness, inadequate social support, poor health status with greater severity of symptoms, rate of progression and coexisting co-morbidities, and poor access to health care [26]. On the other hand, factors like scheduling exercise, motivation, knowledge about exercise, social support, and improvement in health status reinforced long-term adherence [25]. Hence, despite the existence of strong proven benefits of various combination exercise therapies in ideal experimental conditions, to translate such benefits to patients with heart failure with reduced ejection fraction in clinical practice, factors contributing to low patient adherence should be taken into consideration while prescribing an appropriate patient-centered exercise therapy. A randomized control trial on elderly patients with heart failure revealed that physical activity enjoyment completely mediated the relationship between motivation and exercise. Hence, an exercise regimen should be designed in such a way that patients find enjoyment in engaging in activities such as walking, football, modified dancing, or medi-yoga [27]. Home-based telerehabilitation is an effective alternative for patients with low access to facility-based cardiac rehabilitation, as it demonstrated higher adherence rates than control groups [28].

Limitations

Our literature analysis has limitations. We limited our review analysis to English articles published within the last 10 years. We only reviewed free articles, and our study was limited to studies available only in two databases, i.e., PubMed and Google Scholar. More research with a wide network of resources has to be undertaken for specific conclusions.

## Conclusions

Our review emphasizes the benefits and diverse application of resistance training in the cardiac rehabilitation of patients with heart failure with reduced ejection fraction. Through the integration of resistance and aerobic training protocols into the exercise regimen of patients with HFrEF, both vascular reactivity and skeletal myopathy can be addressed, leading to improvements in aerobic capacity and exercise tolerance. Some of the researchers have developed combination therapies that have demonstrated greater improvement in clinical parameters than conventional exercise therapies. Moreover, due to low long-term adherence rates to exercise therapy, prescribing an exercise therapy specific to each patient by considering their preferences is of profound importance. Nevertheless, large-scale RCTs are necessary to design the most effective exercise therapy for the rehabilitation of patients with heart failure with reduced ejection fraction.
